# A Job Demands–Resources Perspective on Kindergarten Principals’ Occupational Well-Being: The Role of Emotion Regulation

**DOI:** 10.3390/ijerph192215030

**Published:** 2022-11-15

**Authors:** Xin Zheng, Qinyuan Dan, Zhimin Wu, Shengquan Luo, Xinying Peng

**Affiliations:** 1 Faculty of Education, Southwest University, Chongqing 400715, China; 2 College of Arts, Chongqing Three Gorges University, Chongqing 404120, China

**Keywords:** emotion regulation, job demands–resources model, occupational well-being, kindergarten principals

## Abstract

The position of school principal is emotionally demanding. Principals’ occupational well-being (OWB) can be influenced by their emotional work characteristics, and their emotional regulation plays a critical role. Based on the job demands–resources (JD-R) model, this study investigated the relationships between kindergarten principals’ OWB and its complex antecedents. Specifically, the study examined the influences among emotional job demands and trust in colleagues on kindergarten principals’ OWB factors (job satisfaction and emotional exhaustion), with a particular focus on the role of their emotion regulation strategies. Through an investigation of 618 kindergarten principals in China, the results showed that emotional job demands and trust in colleagues had different influences on principals’ OWB dimensions. Emotional job demands can enhance both principals’ suppression and reappraisal strategies, and trust in colleagues functions as an interpersonal resource for reappraisal. Principals’ emotion regulation strategies mediated the influence of work characteristics on OWB. Reappraisal is an important personal resource that can buffer the influence of work demands on OWB. The results may extend our understanding of principals’ emotional work. The implications on principals’ work and emotion regulation were further discussed.

## 1. Introduction

Around the world, the push for higher achievement scores has started to cede space to a different set of concerns about students’ and educators’ well-being [1,2]. In comparison to the concern for students’ and teachers’ well-being, leaders’ well-being is overlooked. School principals’ well-being is a determinative factor for teachers’ and students’ well-being. When principals’ well-being declines, their ability to significantly impact school functioning and whole-school well-being also declines [3,4]. Recently, there have been increasing studies concerning primary and secondary school principals’ well-being [3,4], yet few studies have considered principals’ well-being in the early childhood education (ECE) context.

Well-being is a complex construct that involves various aspects, such as professional, social, cognitive and affective well-being [5,6]. Influenced by the affective turn of social science research, the field of educational leadership is shifting to the affective paradigm [7]. In the past, educators’ professional well-being was more focused; in recent years, policymakers have emphasized principals’ emotional well-being [6]. The current study focused on kindergarten principals’ occupational well-being (OWB) from the affective perspective. Current education reform requires principals not only to deal with professional issues but also to lead with emotions [8]. The current COVID-19 pandemic puts higher demands on principals’ jobs, requiring them to be calm and optimistic when facing challenges and fast-changing policies. ECE practice is frequently stated to require a high degree of emotional work [9], and there are few studies exploring the specific effects of emotional work on principals’ emotional well-being [6,9].

To explore how OWB dimensions were influenced by their emotional work environment, the job demands–resources (JD-R) model [10,11] was applied to examine the job’s characteristics and their influences on principals’ OWB. As the model suggested, all types of job characteristics can be divided into two broad categories based on working conditions: job demands and job resources. The JD-R model has been widely adopted to explain how job resources and job demands influence employees’ performance and well-being [11]. It has also been applied to examine the well-being of teachers [12,13] and primary school principals [3] in previous studies. This study specifically examined emotional job demands and social support from colleagues and their influences on principals’ OWB.

Furthermore, there has been increasing focus on the emotional ability of principals’ work, as they need to respond to their own and others’ emotions [3,14,15,16]. Empirical research has begun to elaborate on how principal emotions, as one of the professional capitals in addition to the other three (human, social, and decisional) capitals, add to the leadership repertoire of school principals [4,17,18]. ECE educators are subject to frequent and intense emotional work and kindergarten principals are described as “weather gauge of mood” in kindergartens [9]. Therefore, principals’ emotion regulation ability was considered an important personal resource, which may play a mediating role between job characteristics and OWB.

Thus, the study attempts to explore how emotional job demands and social support from colleagues influence kindergarten principals’ OWB, with a particular focus on the meditating role of emotion regulation. It addresses the following research gaps: it is the first quantitative study that focuses on kindergarten principals’ well-being and its antecedents and consequences. Previous qualitative studies [9] have explored how kindergarten principals regulate their emotions at school, and this study adopted the JD-R model to investigate these complex relationships. Second, the study explored kindergarten principals’ well-being from the emotional perspective, as they experience intense and frequent social interactions with different actors and encounter high emotional demands. The study investigated the relationships among emotional job demands, social support and affective OWB and further analyzed the role of emotional regulation. Third, the study was thus far the first study concerning kindergarten principals conducted in mainland China. As both well-being and emotions are influenced by sociocultural contexts [14,16], the influence of job demands and job resources on emotions and well-being may be attributed to Chinese contextual factors, which could extend our understanding of how school leaders may contribute to the field of principals’ well-being and emotional issues.

## 2. Literature Review

### 2.1. A Job Demands–Resources Model for Principals’ Well-Being

Occupational well-being can be defined as “evaluations of various aspects of one’s job, including affective, motivational, behavioral, cognitive and psychosomatic dimensions” ([5], p. 366). Researchers have increasingly investigated both positive and negative aspects of work emotions at the same time [3,12,19]. In this study, two indicators are used to capture both sides of principals’ well-being: emotional exhaustion reflecting negative emotions stemming from work and job satisfaction reflecting positive thoughts about work. Emotional exhaustion refers to a state of being emotionally drained when one’s emotional resources run out [20]. It is the main aspect of burnout, which may cause people to feel depressed and nervous and thus have a negative impact on their job performance. Job satisfaction, closely associated with people’s psychological well-being [21], is defined as a sense of satisfaction with the work itself [19].

The job demands–resources (JD-R) model [10] has been widely applied to explain the relationships between work environment characteristics and people’s performance and well-being [11]. As mentioned above, two categories of job characteristics were suggested in the model. Job demands (i.e., work pressure and emotional demands from interaction with customers) are defined as physical, psychological, social or organizational aspects of the job that require sustained physical and/or psychological effort and are therefore associated with certain physiological and/or psychological costs [10]. Job resources refer to those physical, psychological, social or organizational aspects of the job that are functional in achieving work goals [11,22]. Job demands have been identified as predictors of exhaustion, psychosomatic health complaints and repetitive strain injury [22,23]. Job resources have been identified as the main drivers of work enjoyment, motivation and work engagement [10,22]. The dual pathways to people’s well-being proposed by the JD–R model have been recognized widely, and the model itself has been proven to predict important organizational outcomes [11].

The JD-R model has been applied in the field of education. For instance, Hakanen, Bakker, and Schaufeli revealed that job demands and job resources were associated with teachers’ burnout and engagement, which further affected their health and organizational commitment. A few exceptions have identified the links between emotional job demands and related psychological effects [21,24,25,26]. Scholars [21,27] have reached a consensus that emotional job demands in a wide range of occupations can lead to ill well-being, such as emotional exhaustion. For principals, Maxwell and Riley found that emotional job demands are positively correlated with primary school principals’ surface acting and deep acting strategies. Emotional job demands may require them to be calm and show care for teachers and students during work, so principals may use emotion regulation to hide their felt emotions and express the unfelt emotions.

Interactions and adaptation to social relationships are essential to understanding emotions [21,28], and thus, the study focused on social support from colleagues as one kind of job resources. Specifically, trust in colleagues, which denotes the quality of relationships among colleagues, is used in this study. Bryk and Schnerider argued that trust relationships are important forms of social capital in schools and are crucial for school development and educators’ well-being [29]. Yin et al.’s study found that teachers’ trust in colleagues can enhance job satisfaction and reduce emotional exhaustion. Based on these studies, principals’ trust in colleagues was hypothesized to fulfill their basic psychological needs, such as relatedness and competence [30], which may promote their satisfaction at work and alleviate their exhaustion.

In sum, emotional job demands were generally correlated with negative well-being factors (e.g., exhaustion), while trust in colleagues serving as a kind of job resource was positively correlated with well-being factors (e.g., satisfaction).

Thus, we hypothesize the following:

**H1.**  *The emotional job demands are positively related to principals’ emotional exhaustion (H1a), but negatively related to their job satisfaction (H1b).*

**H2.**  *Trust in colleagues is negatively related to principals’ emotional exhaustion (H2a), but positively related to their job satisfaction (H2b).*

### 2.2. Emotional Labor and Emotion Regulation

Research on emotion management and displays is found in two fields of research, namely emotional labor (EL) [28] and emotion regulation (ER) [31]. Emotional labor was first defined by Hochschild as “the management of feeling to create a publicly observable facial and bodily display” ([28], p. 7). Two emotional labor strategies were proposed by Hochschild: surface and deep acting [21,28]. Surface acting refers to the strategy by which people suppress their negative emotions or exaggerate their positive emotions to comply with organizational rules, whereas deep acting means that individuals use cognitive techniques to engage in self-paralysis and modify their inner state of emotional experience to meet the demands of the job.

Emotion regulation refers to “the processes by which individuals influence which emotions they have, when they have them, and how they experience and express these emotions” ([31], p. 275). In other words, emotion regulation indicates the individual’s ability to successfully control his or her emotional experiences and expressions [32]. Among the numerous emotion regulation strategies, cognitive reappraisal and expressive suppression are the most identified and discussed [32]. Specifically, cognitive reappraisal is a procedure of cognitive change to reinterpret situational or contextual aspects of stimuli and recognize the situation from a third-person perspective. Conversely, expressive suppression is a form of response modification that inhibits ongoing emotion-expressive behavior [31]. Cognitive reappraisal happens earlier than expressive suppression, so it may be more beneficial to change emotion than expressive suppression, which “consumes cognitive resources, impairing memory for information presented during the emotion regulation period” ([32], p. 289).

Both emotion regulation and emotional labor capture critical aspects of emotion management, and research findings on emotion regulation and emotional labor in the workplace are ample [27,33]. Some researchers [21,27,34] have proposed integrating the two research traditions. Grandey connects the two emotion regulation strategies to deep and surface acting by employees interacting with the public or clients. Deep acting is mapped onto reappraisal as an antecedent-focused emotion regulation strategy or a means to change one’s mood and expressions and exhibit a positive affect to customers. Surface acting is mapped onto suppression as a type of response-focused emotion regulation, where felt emotions are modified in terms of how they are expressed or acted on. Mikolajczak et al. discuss how the most common regulation strategies in the model of emotion regulation are connected with emotional labor [34]. For example, deep acting can be accomplished by emotion regulation strategies such as distraction, situation modification and positive reappraisal. As Grandey argued, “both ER and EL researchers are interested in modifying feelings…and modifying expression…when interacting with others” ([35], p. 55).

Emotion is inherent to the practice of leadership [15], and “leadership work is hard emotional labor” ([36], p. 137). The centrality of emotions in the personal and professional practice of school leaders is by now well-established in scholarship [15,36]. Principals are supposed to manage and regulate their emotions continually for schooling and teaching processes containing many emotions [14,15,37]. Specifically, for the proper functioning of the school system, principals need to establish or maintain cooperative and harmonious relations with other individuals, and they use a variety of strategies to appear self-controlled [14], which may require them to undertake many emotional endeavors. Principles were found to employ an array of emotional strategies to manage the self and others in interactions ([36], p. 142). They showed that the idea of ensuring children’s safety and creating a positive emotional climate in the school was the most important aspect of the leaders’ visions for the schools. This focus places the creation of a positive emotional culture at the center of leadership practice in schools [36]. Crawford found that principals need to regulate their own emotions and manage the emotions of staff in their efforts toward emotional coherence. To and Yin found that kindergarten principals in Hong Kong utilize emotion regulation strategies to help the kindergarten succeed through social interactions. Oplatka’s study in Israel showed that school principals are allowed, even encouraged, to display cognitive, affective and behavioral empathy while consciously inhibiting the expression of anger and fear publicly under certain circumstances [16].

In other words, principals are currently confronted with many sensitive demands, such as the needs of relevant stakeholders, competing objectives, tricky stakeholder interactions, the impression others have of them and teachers’ emotions [4,14], which require them to make use of emotion regulation or emotional labor strategies to perform their duties. Despite the fact that ‘‘interest in educational leaders and emotions seems to be in its early stages’’ ([14], p. 158), clarifying principals’ emotion regulation and its antecedents and consequences is necessary.

### 2.3. Emotion Regulation as Personal Resource in the JD-R Model

It is not surprising that even if facing the same job demands and possessing the same job resources, different people may behave and feel very differently in similar working conditions [11,38]. Therefore, the recent JD-R model added personal resources to the model [11,38], which emphasized the role of individual agency and resilience in modifying the effects. Personal resources are “aspects of the self that are generally landed to resilience and refer to individuals’ sense of their ability to control and impact upon their environment successfully” ([38], pp. 123–124), which can be important determinants of their adaptation to work environments [38]. Following this theoretical implication, the study considers principals’ emotion regulation as a kind of personal resource that plays a mediating role between job demands, job resources and OWB factors. Some researchers regard emotion regulation as a mediator that plays a mediating role between job characteristics and teachers’ well-being [12].

Previous studies have documented the complex relationships between emotional job demands, trust in colleagues, ER and OWB dimensions, which laid the foundations for hypothesizing ER as a mediator. Maxwell and Riley found that primary and secondary school principals’ emotional demands could enhance both surface acting and deep acting [3]. Other qualitative studies have described how emotional demands and rules are related to principals’ ER or emotional labor [15,16,36]. For example, principals are asked to demonstrate rational and positive selves, which requires them to reappraise the actual situation to adjust their feelings or even suppress what they truly feel [15]. Therefore, emotional job demands lead principals to use either reappraisal or suppression strategies to show an affable and proactive emotion. Regarding trust in colleagues and ER, studies have demonstrated the beneficial role of a trustful environment in schools for improving teachers’ resilience ability and professional competence [12,39]. In a trusting environment, principals are found to use more deep acting [40] and express authentic emotion more often [12].

Thus, we proposed the following hypotheses:

**H3.**  *The emotional job demands are positively related to reappraisal (H3a) and suppression (H3b).*

**H4.**  *Trust in colleagues is positively related to reappraisal (H4a) but negatively to suppression (H4b).*

Furthermore, the relationship between ER and OWB factors was also analyzed in previous studies. Generally, suppression was correlated with negative aspects of well-being, while reappraisal led to positive influences on well-being. Gross and John found that the frequent use of suppression is related to ill well-being [41]. Individuals who reported suppressing their felt emotions and showed inauthentic display of emotion felt dissatisfied both with themselves and their relationships, indicating that they were emotionally exhausted [42,43]. Therefore, suppression has been identified to have an adverse effect on an individual’s health [27,44] and job satisfaction [33,45]. Reappraisal is expected to promote well-being because one of its main functions is to diminish the perception of adversity early in the emotion process [41,42,46]. In general, reappraisal is more positively associated with well-being than suppression [41,47,48].

Thus, we hypothesize the following:

**H5.**  *Suppression is positively related to emotional exhaustion (H5a) but negatively related to job satisfaction (H5b).*

**H6.**  *Reappraisal is negatively related to emotional exhaustion (H6a) but positively related to job satisfaction (H6b).*

**Figure 1:**
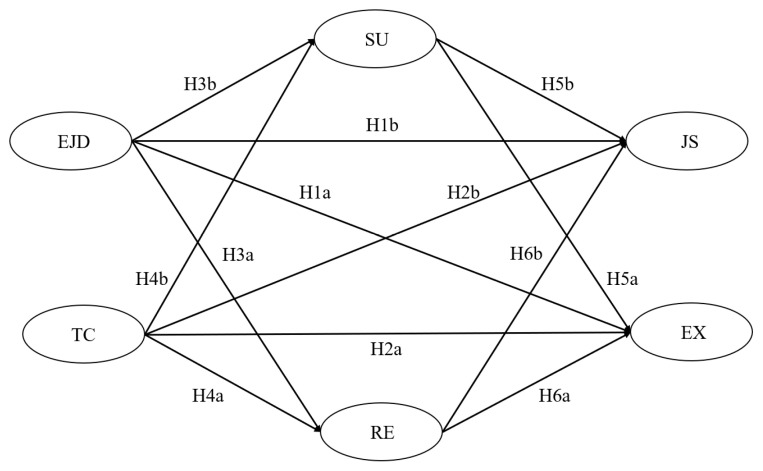
The hypothetical model. Note: EJD = emotional job demands, TC = trust in colleagues, RE = reappraisal, SU = suppression, EX = emotional exhaustion, JS = job satisfaction.

In summary, emotion regulation strategies are added to the JD-R model as a personal resource to test their mediating effects on the relationships between emotional job demands, trust in colleagues and principals’ OWB. Using a sample of kindergarten principals, the study examined (a) the relationships between two characteristics of principals’ work environment, i.e., emotional job demands and trust in colleagues, and two well-being indicators, i.e., emotional exhaustion and teaching satisfaction, and (b) the mediating role of two emotion regulation strategies (i.e., reappraisal and suppression) in the relationships between job characteristics and principals’ well-being. The hypothetical model of the study is shown in Figure 1.

## 3. Methodology

### 3.1. Participants

A self-reported questionnaire survey was conducted for data collection from July to October 2021, when principals participated in provincial-wide professional development training in universities in northern China. In China, both novice and experienced principals are required to participate in different levels of training programs, which are mainly provided by local colleges and universities. The data were collected from six universities. Through the random sampling strategy, we distributed 700 questionnaires to the participants in the universities. Eventually, a total of 618 valid copies were collected for this study. All kindergarten principals were voluntary participants in the survey.

The sample comprised 618 kindergarten principals. There were 62 males (10%) and 556 females (90%), including 180 (29.1%) under the age of 30, 241 (39%) between the ages of 30 and 40, 145 (23.5%) between the ages of 40 and 50, 21 (3.4%) over the age of 50 and 31 participants who did not report their age. In terms of their academic qualifications, 4 (0.6%) had a graduate degree, 357 (57.8%) had a bachelor’s degree, 215 (34.8%) had a college degree and 4 (0.6%) had a high school and subsequent degree. Regarding their working experience as principals, 387 (62.6%) had worked for 5 years or less, 88 (14.2%) had worked for 6–10 years, 69 (11.2%) had worked for 11–20 years, 143 (2.1%) had worked for 21 years or more and 61 participants did not report their working years. Among these principals, 509 (82.4%) had teaching tasks, and 109 (17.6%) had no teaching tasks.

In view of the schools where they work, 343 (55.5%) were rural schools, 192 (31.1%) were country and town schools and 83 (13.4%) were urban schools. Among them, 451 (73%) were public schools, and 167 (27%) were private schools. Regarding their schools’ overall ranking of student enrollment quality in the region, 286 (46.3%) were in the top 10%, 92 (14.9%) were in the top 10–30%, 106 (17.2%) were in the top 60–80% and 17 (2.8%) were in the bottom 20%.

### 3.2. Instruments

#### 3.2.1. Emotional Job Demands and Trust in Colleagues

The emotional job demands scale (EJDS) [49] was used to examine kindergarten principals’ job demands. This scale consists of four items that mainly assess principals’ perceptions of the emotional demands of their work. Examples of these items include “I have to use my emotions and behaviors to create a reassuring climate for my students and colleagues”. The participants rated each item on a 5-point Likert scale ranging from 1 (strongly disagree) to 5 (strongly agree).

The trust in colleague scale (TICS) was adapted by Yin and his colleagues from Tschannen-Moran [50] to examine kindergarten principals’ trust in their colleagues. It contains five items that assess the five facets of trust between principals and their colleagues: benevolence, reliability, honesty, competence and openness. Examples of these items include “Teachers in this school have faith in the integrity of their colleagues”. The participants rated each item on a 6-point Likert scale ranging from 1 (strongly disagree) to 6 (strongly agree).

#### 3.2.2. Emotion Regulation Strategies

The emotion regulation questionnaire (ERQ) [41] was used to examine kindergarten principals’ usage of cognitive reappraisal and suppression strategies. Six items assessed reappraisal (e.g., “When I’m faced with a stressful situation, I make myself think about it in a way that helps me stay calm.”), and four items assessed suppression (e.g., “I control my emotions by not expressing them.”). The participants rated each item on a 5-point Likert scale ranging from 1 (strongly disagree) to 5 (strongly agree).

#### 3.2.3. Kindergarten Principals’ Well-Being

The five items of the Maslach burnout inventory—general survey (MBI) [20] were used to examine kindergarten principals’ emotional exhaustion. Examples of these items include “I feel emotionally exhausted because of my work.” The participants rated each item on a 5-point Likert scale ranging from 1 (strongly disagree) to 5 (strongly agree).

The job satisfaction scale (JSS) [51] was used to examine kindergarten principals’ job satisfaction. This is a single-factor, 5-item scale. Examples of these items include “In most ways, being a principal is close to my ideal”. The participants rated each item on a 5-point Likert scale ranging from 1 (strongly disagree) to 5 (strongly agree).

All six scales were translated and administered in the Chinese language. Furthermore, convergent validity, criterion-related validity and incremental validity have been confirmed by previous studies [12].

### 3.3. Analysis

SPSS 21.0 and Mplus 8.0 were used to conduct the analyses. The descriptive statistics and correlations were calculated by SPSS. Then, structural equation modeling (SEM) was conducted by using Mplus. SEM was used to examine the relationships between the constructs of interest. SEM can be used to assess unobservable latent constructs defined by one or more observed variables and to model all of the parameters simultaneously. Confirmatory factor analysis (CFA) was used to examine the construct validity of the scales.

The model fit was assessed by using the chi-square value (χ^2^), the root mean square error of approximation (RMSEA), the Tucker–Lewis index (TLI) and the comparative fit index (CFI). The data fit is acceptable when CFI and TLI are no less than 0.90 (the higher, the better), and an acceptable fit requires the RMSEA to be under 0.10 (the lower, the better) [52]. For the mediation analysis, a bootstrap approach was used to detect indirect effects [53].

## 4. Results

### 4.1. Reliability and Construct Validity of the Scales

The Cronbach’s alpha (α) of the scales ranging from 0.70 to 0.95, indicating sound internal consistency. CFA was conducted to test the construct validity of each scale. The results showed acceptable data fit for EJDS (χ^2^ = 8.92, df = 2, *p* < 0.001, RMSEA = 0.07, CFI = 0.96, TLI = 0.90) and TICS (χ^2^ = 8.80, df = 5, *p* < 0.001, RMSEA = 0.04, CFI = 0.99, TLI = 0.98). Additionally, we used a second-order factor structure for ERQ, and the results indicate that the structure had acceptable data fit (χ^2^ = 292.6, df = 34, *p* < 0.001, RMSEA = 0.09, CFI = 0.92, TLI = 0.90). Further, both the JSS (χ^2^ = 31.72, df = 5, *p* < 0.001, RMSEA = 0.09, CFI = 0.96, TLI = 0.93) and emotional exhaustion scale (χ^2^ = 32.30, df = 5, *p* < 0.001, RMSEA = 0.10, CFI = 0.98, TLI = 0.95) showed acceptable data fit. These results showed that all the scales had good internal consistency and construct validity.

### 4.2. Descriptive Results

The descriptive results, correlations and reliabilities are presented in Table 1. Kindergarten principals scored highest in their perception of trust in colleagues (mean (M) = 5.62, standard deviation (SD) = 0.52) and lowest in their strategies of emotional exhaustion (M = 2.41, SD = 1.23). For the correlations, except for the nonsignificant relationships between emotional exhaustion and both job demands and job satisfaction, all of the variables significantly correlated with each other.

These correlations followed the definitions of these variables, providing preliminary evidence for the construct validity of the scales.

### 4.3. SEM Results

**Figure 2:**
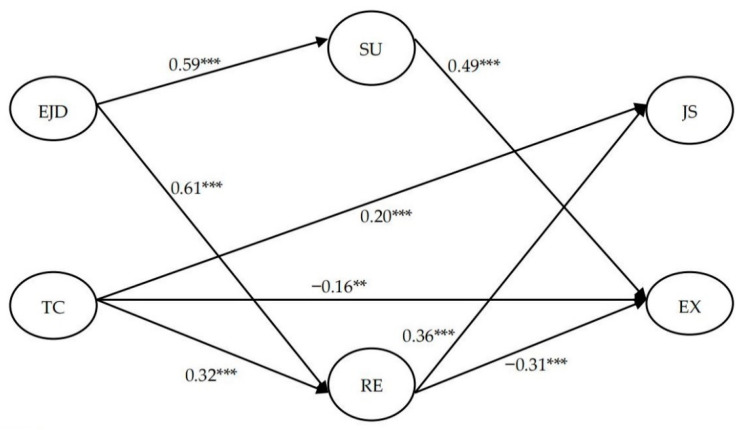
Path analysis results of the hypothesized model (showing significant paths only). Note: ** *p* < 0.01, *** *p* < 0.001. EJD = emotional job demands, TC = trust in colleagues, RE = reappraisal, SU = suppression, EX = emotional exhaustion, JS = job satisfaction.

We used regression analysis to explore the influences of demographic variables (gender, age, working age as principals and degree) on OWB dimensions. The results showed that gender, working years and degree had no significant influences on job satisfaction. Principals’ age, working years and degree had no significant effects on their emotional exhaustion. Gender (1 = male, 2 = female) was found to significantly influence emotional exhaustion (*β* = −0.50, *p* < 0.01) and age influenced principals’ job satisfaction (*β* = −0.12, *p* < 0.05). Then, according to the purpose of the study, we tested the structural model to explore the relationships between kindergarten principals’ job characteristics, emotion regulation strategies and well-being indicators. The results are displayed in Figure 2. The results indicated an acceptable fit between our theoretical model and the data (χ^2^ = 1519.457, *df* = 367, *p* < 0.001, RMSEA = 0.07, CFI = 0.92, TLI = 0.91).

The SEM results indicated that significant associations existed between job demands and the two emotion regulation strategies. Specifically, emotional job demands were positively associated with suppression (*β* = 0.59, *p* < 0.001) and reappraisal (*β* = 0.61, *p* < 0.001), supporting H3. Trust in colleagues was positively related to reappraisal (*β* = 0.32, *p* < 0.001), so H4a was supported while H4b was not. In addition, trust in colleagues was found to be negatively related to emotional exhaustion (*β* = −0.16, *p* < 0.01) but positively related to job satisfaction (*β* = 0.20, *p* < 0.001), so H2 was supported while H1 was not.

Furthermore, suppression was positively associated with emotional exhaustion (*β* = 0.49, *p* < 0.001), so H5a was supported while H5b was not. Reappraisal was found to be positively associated with job satisfaction (*β* = 0.36, *p* < 0.001) but negatively related to emotional exhaustion (β = −0.31, *p* < 0.001), supporting H6.

### 4.4. Mediation Results

The mediation effects were tested based on 1000 bootstrap samples, and the results are summarized in Table 2. The effect size of the mediator was reported by using the point estimate of the indirect effect. As Hayes suggests, the indirect effect is significant if zero is not between the lower and upper bounds in the 95% confidence interval.

Specifically, the results showed that reappraisal significantly mediated the effect of emotional job demands on emotional exhaustion (*β* = −0.19, *p* < 0.01) and job satisfaction (*β* = 0.22, *p* < 0.001). Suppression significantly mediated the effect of job demands on emotional exhaustion (*β* = 0.29, *p* < 0.001), while reappraisal significantly mediated the effect of trust in colleagues on job satisfaction (*β* = 0.11, *p* < 0.001).

## 5. Discussion

Using the JD-R model, the study examined kindergarten principals’ emotional job demands, trust in colleagues and their influences on emotion regulation and OWB factors. It uncovered the complex relationships between antecedents and consequences of emotion regulation in the Chinese kindergarten context. The results highlighted the influences of emotional job demands and trust in colleagues and principals’ emotion regulation as an important personal resource at work.

### 5.1. Influences of Job Resources and Job Demands on Principal Emotion Regulation and OWB

The work characteristics were categorized as emotional job demands and trust in colleagues in this study, and these two kinds of elements showed different influences on principals’ ER and OWB. Regarding trust in colleagues, the association between trust in colleagues and an employee’s well-being has been established in previous research [12,39]. The results showed that trust in colleagues can significantly enhance job satisfaction (H2b) and reduce emotional exhaustion (H2a). This is not surprising since a trustful relationship is beneficial for principals to meet their basic psychological needs [30,50], which may promote principals’ perception of satisfaction at their work and eliminate the sense of emotional exhaustion. Our research indicated that Chinese kindergarten principals’ job satisfaction was positively linked with their sense of trust, meaning that in a trusting work environment, kindergarten principals are more likely to enjoy their work and feel more energetic and satisfied with the job.

In addition, trust in colleagues can positively predict principals’ reappraisal strategy (H4a). When principals perceive that their colleagues are more reliable, competent, honest and open, they tend to use their reappraisal strategy more to regulate their emotions. Through sharing their emotions with other trusted colleagues or close friends, principals find a way to release negative emotions [15,36], and they tend to interpret the situation from a perspective of goodwill rather than inhibiting their experience and expression of negative emotions. Crawford’s case study showed that when principals experienced certain emotions (e.g., anger), these feelings needed somewhere to go, and she mentioned “talking it through with her senior management team” ([15], p. 527). To and Yin’s study revealed an interpersonal emotion regulation strategy, which refers to the process whereby principals change their feelings through social interactions. For example, principals turn to others (colleagues) for comfort and sympathy. Kindergarten colleagues were an important resource for principals to “share the sorrow and joys with”, and specific colleagues who can always be trusted by the principals are essentially valued as they provide a sense of security ([9], p. 322).

Emotional job demands were found to positively predict both suppression and reappraisal (H3a, H3b), which is slightly different from previous studies, as most previous studies concerned the negative role of job demands at work [3]. Interestingly, in our research, we found that Chinese kindergarten principals apply both reappraisal and suppression strategies when facing emotional job demands. In other words, when facing higher emotional job demands, principals either use a reappraisal strategy or suppression strategy at the same time to keep calm and show control of their emotions. There may be two possible reasons. First, the creation of a positive emotional culture was placed at the center of leadership practice in schools [15,36]. Principals regulate their own emotions and manage the emotions of staff in their efforts toward emotional coherence [15]. Principals actively engage in emotional sensitizing of the staff and children [36], such as setting the emotional tone within the school, reminding teachers that they cannot be so hard on children, and giving them breaks. These reappraisal strategies are commonly used in principals’ work due to the high expectations of colleagues, parents and the public [3,15,16]. At the same time, principals were also found to restrain and suppress their emotions at work [9]. School principals need to create a safe school and a positive emotional climate in the school at all times [15,36]. Second, some cultural factors may contribute to the results, such as leaders being role models and maintaining harmony. In Chinese traditions and cultures, the leader of an organization was seen as a model in every aspect [54,55], including emotional aspects. Leaders should always remain calm and stable in carrying out their duties and model their emotions in front of children and staff [9,54]. To and Yin used the metaphor of the “weather gauge of mood” to denote kindergarten principals’ emotion regulation, since “principals are like a gauge that is checked by kindergarten staff and which influences them, careful management of emotions to show desirable displays is needed” ([9], p. 324). In addition, Matsumoto et al. argued that in cultures emphasizing the maintenance of social order, people tend to use suppression strategies, and suppression may be positively associated with reappraisal, which is consistent with our findings [56].

### 5.2. Mediation of Principals’ Emotion Regulation

Regarding the effects of emotion regulation on OWB, the results showed that suppression and reappraisal had the reverse influence on OWB. Specifically, suppression enhances emotional exhaustion (H5a), while reappraisal is positively linked to job satisfaction (H6b) and negatively linked to emotional exhaustion (H6a). The results were consistent with previous studies concerning emotion regulation and well-being factors [41,57]. Generally, suppression or surface acting was positively related to emotional exhaustion. People using suppression tend to be less satisfied with their lives, less optimistic and have higher levels of stress [16,42,48]. Because suppression operates later in the emotion sequence, expressive suppression is much less efficient in reducing the negative emotional experience and protecting against adversity and promoting wellness [41,48]. Principals who suppress their feelings more tend to have higher levels of emotional exhaustion. Even though previous qualitative studies have well-documented how principals can effectively suppress or not display certain emotions at work [15,16,36], our results showed that such a strategy may result in emotional exhaustion in the long run.

Reappraisal is expected to promote well-being because one of its main functions is to diminish the perception of adversity early in the emotion process [41,42,46]. In general, reappraisal is more positively associated with well-being than suppression [41,47,48]. Previous qualitative studies revealed that principals adopted more reappraisal strategies due to the public expectations and colleagues’ needs and their responsibilities they take [15,16,36]. When principals report more frequent reappraisal strategies, they can better use their cognition, control and reflect on the environment, which might lead to a higher level of well-being. Our results showed that reappraisal is not only an effective strategy for school management, but also an important resource for principals’ satisfaction.

Furthermore, our research also found a mediating role of emotion regulation strategies in job characteristics and OWB. Specifically, suppression mediated the influence of emotional job demands and emotional exhaustion. Reappraisal significantly mediated three paths (EJD-EX, EJD-JS, TC-JS). The results were similar to previous studies involving teachers. For example, Yin et al. investigated 1115 primary school teachers in Hong Kong and showed that both emotion regulation strategies mediated the relationships between both emotional job demands and trust in colleagues and teachers’ well-being. Our research is based on the perspective of Chinese kindergarten principals, who are relatively neglected by researchers in the field of emotion regulation. Specifically, emotional job demands can directly positively influence principals’ emotional exhaustion via suppression. Conversely, via the indirect effect of reappraisal, emotional job demands are significantly associated with job satisfaction and negatively associated with emotional exhaustion.

Based on these findings, the results showed that emotion regulation strategies, especially reappraisal, were an important personal resource to buffer the influence of job demands and to facilitate the influence of trust relationships. In the JD-R model, personal resources refer to individuals’ sense of their ability to control and impact their environment [38]. Reappraisal strategies, such as attention deployment and cognitive change, may not change the external environment, but they change how individuals interact with the external environment and different actors. Such emotional ability is an important capital for educators [18,36]. When facing the same job characteristics, principals who have a higher level of reappraisal ability may be psychologically healthier than those who suppress true feelings. Therefore, in their daily work, instead of keeping their emotions within, principals need to change their mindset and reappraise the situation to turn the disadvantages into advantages. The results, based on quantitative analysis, further proved the importance of interpersonal emotion regulation, as previous qualitative studies revealed [9,15].

## 6. Implications for Practice

The study provides several implications for related educators and policymakers.

First, there should be more attention paid to the emotional aspects of kindergarten principals, as they were often seen as a silent or dark side of leadership work [9,58]. School principals currently encounter considerable job demands, such as diverse tasks managing staff, organizing budgets and providing strategic organizational focus alongside high-stakes testing [3]. Coping with these rational issues requires demanding emotional work. Additionally, the emotional side of the principal’s work was even described as the “dark side” [58]. Expressing emotions was seen as less professional and less rational in schools dominated by current values of professionalism [37]. Our quantitative study showed that kindergarten principals’ emotional job demands are high, and they may influence principals’ well-being. In current principal preparation or professional development programs, we should go beyond the mere “rationality” of leadership, as both rational and emotional aspects of principals’ work are equally important in leading schools successfully [15,16,59].

Second, principals’ emotion regulation strategies were significantly related to their OWB. For example, suppressing their own emotions is frequent [15,16,36]; however, even though this strategy is sometimes useful in managing school staff, it may be detrimental for their well-being in the long run. Notably, reappraisal is a useful emotion regulation strategy for principals. It can not only bring about positive OWBs but can also buffer the influence of increasing job demands. When facing higher job demands, reappraisal strategies such as attention deployment and cognitive change can help them better deal with the negative influences of job demands. More programs that concern leaders’ emotion regulation and other emotion-related problems could be provided. Principals need to learn how to deal with increasing job demands and the frequent and intense interpersonal relationships within and outside their schools or kindergartens.

Third, fostering a trustful and supportive atmosphere in kindergartens is not only beneficial for teachers but also healthy for the principals themselves. Previous studies have well-documented that establishing trusting relationships is a core leadership practice of school leaders [58,60]. Our study shows that in a trustful school environment, kindergarten principals are more likely to use reappraisal strategies, which could further enhance their satisfaction. In a supportive and trusting climate, principals can turn to their colleagues (e.g., teachers or middle leaders) for help, and such interpersonal ER strategy has been found to be significant [9,15,36]. Therefore, kindergarten principals are supposed to make efforts to establish trusting relationships with colleagues and create a climate of authenticity in kindergartens, especially in high-power distance societies where the leaders of the organizations have much power in determining the atmosphere [55,61]. Principals need to take action first and show their openness to colleagues [62].

Several limitations should be noted in this study. First, the sample size is still small compared to the large number of kindergartens in China. Future studies can improve the sample size by adopting multiple sampling strategies, such as a stratified sampling strategy. Second, common method biases arise from having a common rater, a common measurement context or a common item context [63]. The data were collected by principals’ self-report questionnaires, and only Likert rating scales were used to evaluate principals’ perception of their well-being, emotion regulation and their working environment. Thus, the results may suffer from common-method bias. Further research could use more data sources (e.g., observations, interviews) to obtain more objective data about principals’ emotions. Multiple data sources can also help to analyze the complex and dynamic relationships between principals’ well-being and their working environment. Qualitative studies can be conducted to explore how principals regulate their emotions in response to their job demands and how social support from colleagues can influence their emotions and well-being. Third, this cross-sectional design made it impossible to determine causal relationships between the variables in the study. Longitudinal studies are encouraged to determine the causal status of these relationships.

## 7. Conclusions 

By clarifying the antecedents and consequences of Chinese kindergarten principals’ emotion regulation using the JD-R model, the results showed that emotional job demands can positively influence both principals’ use of suppression and reappraisal strategies. Trust in colleagues as a kind of job resource can facilitate principals’ reappraisal and job satisfaction and reduce their emotional exhaustion. Principals’ emotion regulation is an emotional resource that can mediate the relationship between job demands, trust in colleagues and OWB factors. These findings enrich the understanding of kindergarten principals’ work and their OWB through the emotional perspective.

## Figures and Tables

**Table 1:** Means, standard deviations, bivariate correlations and reliability measures of all of the variables (N = 618).

Variables	Mean	SD	1	2	3	4	5	6
1. Emotional job demands	4.32	0.64	(0.82)					
2. Trust in colleagues	5.62	0.52	0.39 **	(0.95)				
3. Reappraisal	4.44	0.51	0.60 **	0.55 **	(0.86)			
4. Suppression	3.91	0.80	0.42 **	0.21 **	0.49 **	(0.70)		
5. Emotional exhaustion	2.41	1.23	0.02	−0.21 **	−0.13 **	0.21 **	(0.91)	
6. Job satisfaction	4.29	0.70	0.36 **	0.41 **	0.45 **	0.21 **	−0.07	(0.94)

Note: ** *p* < 0.01; SD = standard deviation; Cronbach’s α in parentheses along the diagonal.

**Table 2:** Mediation of emotional regulation strategies on the relationship between job characteristics and principals’ well-being.

Independent Variable	Dependent Variable	Mediation Analysis			
		Mediation Variable	Estimates (SE)	*p*	95% CI
EJD	EX	SU	0.29 (0.07)	0.000	[0.34, 0.88]
		RE	−0.19 (0.06)	0.003	[−0.73, −0.18]
	JS	RE	0.22 (0.04)	0.000	[0.13, 0.31]
TC	JS	RE	0.11 (0.03)	0.000	[0.09, 0.27]

Note: EJD = emotional job demands, TC = trust in colleagues, RE = reappraisal, SU = suppression, EX = emotional exhaustion, JS = job satisfaction.
